# Simultaneous Extraction and Identification of Phenolic Compounds in *Anoectochilus roxburghii* Using Microwave-Assisted Extraction Combined with UPLC-Q-TOF-MS/MS and Their Antioxidant Activities

**DOI:** 10.3389/fpls.2017.01474

**Published:** 2017-08-24

**Authors:** Mengjie Xu, Qingsong Shao, Shenyi Ye, Shuailing Li, Mei Wu, Mozhi Ding, Yanjing Li

**Affiliations:** ^1^State Key Laboratory of Subtropical Silviculture, Zhejiang A & F University Hangzhou, China; ^2^The Department of Traditional Chinese Medicine, Zhejiang A & F University Hangzhou, China; ^3^Jinhua Academy of Agricultural Sciences Jinhua, China; ^4^Jinhua Jinglong Biological Technology Co., Ltd. Jinhua, China; ^5^Zhejiang Jiangkang Agricultural Science and Technology Co., Ltd. Jinhua, China

**Keywords:** *Anoectochilus roxburghii*, microwave-assisted extraction, response surface methodology, phenolic compounds, antioxidant activity

## Abstract

This study used MAE and RSM to extract phenolic compounds from *Anoectochilus roxburghii*, and the optimum conditions defined by the model to give an optimum yield of 1.31%. The antioxidant activity *in vitro* showed when the concentration of phenolic compounds was reached 1 mg mL^-1^, the clearance rates were 82.58% for DPPH and 97.62% for ABTS^+^. *In vivo* antioxidant experiments used D-galactose to build oxidative damage in healthy Kunming mice. The result showed that the extractions of *A. roxburghii* can improve the antioxidant ability and the medium and low dose groups had better ability to scavenge free radicals. The UPLC-Q-TOF-MS/MS was developed to identify 21 kinds of phenolic compounds by molecular mass, ms/ms fragmentation, as well as retention time. The result showed that the phenolic compounds of *A. roxburghii* had significant potential as a natural antioxidant to promote health and to reduce the risk of disease.

## Introduction

Oxidative stress, a result of imbalance between the antioxidant defense system and the formation of reactive oxygen species (ROS), may induce damage to cellular biomolecules such as proteins, enzymes, carbohydrates, and lipids through oxidative modification and contributing to the pathogenesis of human diseases. However, many synthetic antioxidants, such as butylated hydroxytoluene (BHT), butylated hydroxyanisole (BHA), tertbutylhydroquinone (TBHQ) and propyl gallate (PG), may accumulate in the body, resulting in liver damage and carcinogenesis (Mukhopadhyay et al., 2006; Galano et al., 2011). On account of this, more attention has been paid to extract natural non-toxic antioxidants in an effort to protect the human body from free radicals and retard the progress of many chronic diseases (Adams and Ghaly, 2007). Phenolic compounds are an important group of natural antioxidants that are distributed in medicinal plants, vegetables, fruits, and spices. Most phenolic compounds reported to be present in plants are quercetin, gallic acid, catechin, galuteolin, chlorogenic acid, ferulic acid, and kaempferol. Many researchers have shown much great interest in natural products because of their safety and their wide acceptance by consumers (Ranic et al., 2014).

*Anoectochilus roxburghii* is a perennial herb of the Orchidaceae family, which is mainly distributed in Southern China, Japan, India, Sri Lanka, Nepal, and Southeast Asian countries (Lv et al., 2015). Because of its unique medicinal properties, *A. roxburghii* has been called “the king of medicines” in China (Du et al., 2008; Cui et al., 2013; Shao et al., 2014). It has been used to treat diabetes, tumors, hyperliposis, and hepatitis. The studies on the chemical compounds of *A. roxburghii* were conducted, including phenolic, polysaccharides, triterpenoids, amino acids, etc. In preliminary study, we confirmed that *A. roxburghii* was rich in phenolic compounds. Phenolic compounds were considered to have many functions, such as removing active oxygen, preventing hemal sclerosis, improving nutrition for tissue, antiaging, and preventing aging dementia (Xi and Wang, 2013; Chen et al., 2015). However, few studies reported the extraction process optimization and antioxidant activity of *A. roxburghii* systematically. Thus, this study contains three objectives (1) to optimize the microwave-assisted extraction (MAE) process by response surface methodology, (2) to identify the phenolic compounds from *A. roxburghii* by UPLC-Q-TOF-MS/MS, and (3) to explore the antioxidant activity of the extractions of *A. roxburghii* and explored the mechanism of the antioxidant defense system.

## Materials and Methods

### Plant Materials and Chemicals

*Anoectochilus roxburghii* samples were identified by Professor Runhuai Hu at Zhejiang Agriculture and Forestry University, Lin’an City, Hangzhou, Zhejiang Province. The fresh herb of *A. roxburghii* were dried in a blast oven at 60°C to a stable moisture content of less than 5%, processed in a high-speed rotary cutting mill, and finally screened to produce a fraction containing particles as large as 100 mesh (149 μm). The powder was stored at 4°C in airtight bags until analysis. Total superoxide dismutase (T-SOD), glutathione peroxidase (GSH-Px), malondialdehyde (MDA), and commercial protein assay kits were obtained from Nanjing Jiancheng Bioengineering Institute (Nanjing, China). Folin–Ciocalteu reagent was obtained from Solarbio (Beijing, China). Gallic acid, 2,2-diphenyl-1-picrylhydrazyl radical (DPPH), and 2,2′-azinobis-(3-ethylbenzthiazoline-6-sulfonate) (ABTS) were obtained from Sigma-Aldrich. Chlorogenic acid, caffeic acid, L-epicatechin, ferulic acid, rutin, luteolin, quercetin, kaempferol, and apigenin were purchased from the National Institute for the Control of Pharmaceutical and Biological Products (Beijing, China). Acetonitrile and acetic acid of HPLC grade were purchased from Fisher Scientific (Pittsburgh, PA, United States). Ultrapure water was purified with a Milli-Q system (Millipore, Billerica, MA, United States). All other chemicals were of analytical grade and were obtained from Sinopharm Chemical Reagent, Co. Ltd (Shanghai, China).

### Extraction Process Optimization

A portion of *A. roxburghii* powder was carefully weighed (1.0 g) and added to a 150 ml round-bottom flask. The flask was placed in the microwave apparatus and was fitted with a water condenser. The method analyzed four factors of extraction time (5–65 min), microwave power (100–500 W), solid–liquid ratio (1:40 to 1:80), and ethanol concentration (65–85%). After extraction, the supernatant was collected and the residue was extracted again. The combined supernatants were concentrated in a rotary evaporator, and stored at 4°C for later use. Response surface methodology was used to determine the optimal combination of process parameters, which varied on four factors and five levels. The selected extraction time, microwave power, solid–liquid ratio, and ethanol concentration were used as independent variables and total phenolics yield was used as a response value in a response surface experiment. The experimental data were analyzed using Design-Expert 8.0.6 trial software and determine the best microwave extraction conditions for total phenolics of *A. roxburghii.*

### Antioxidant Activity *In Vitro*

The DPPH radical scavenging activity of the *A. roxburghii* extracts were determined according to the literature method (Dolinsky et al., 2015) with minor modifications. VC standard and sample solutions were prepared in a concentration series. The three tubes were marked A, B, and C. Group A: 2 mL of sample (VC) solution and 2 mL of DPPH solution; Group B: 2 mL of sample (VC) and 2 mL of absolute ethanol; Group C: 2 mL of 50% ethanol and 2 mL of DPPH solution. Sample (VC) is same for group A and group B. The reaction liquid was placed in darkness at room temperature for 30 min, and the absorbance at 517 nm was measured (*n* = 3).

The antioxidant activities of *A. roxburghii* extracts against the stable ABTS radical cation were determined according to the method of Shao et al. (2014) with slight modification. ABTS standard solution (7 mmol L^-1^) and potassium persulfate solution (140 mmol L^-1^) were prepared separately. ABTS solution (5 ml) was mixed with potassium persulfate solution (88 μL) and placed in darkness for 12–16 h. An ABTS working solution was also prepared by diluting the ABTS solution with ethanol to adjust the absorbance to 0.7 at 734 nm. Samples and VC reference standard were prepared at various concentrations and were to tubes (A, B, or C). Tube A contained sample solution (1 mL) or VC solution (1 mL) and ABTS working liquid (3 ml); tube B tube contained sample or VC solution (1 mL) and anhydrous ethanol (3 mL); tube C contained sample or VC solution (1 mL) and ABTS working solution (3 mL). The reaction liquid was placed in darkness at room temperature for 20 min, and the absorbance at 734 nm was recorded (*n* = 3). The antioxidant activity was calculated according to the equation as follow: where *A*_0_ is the absorbance of group C; *A*_1_ is the absorbance of group A; *A*_2_ is the absorbance of group B.

$$\%\text{Inhibition}=\frac{A_{0}-A_{1}+A_{2}}{A_{0}}\times 100$$

### Antioxidant Activity *In Vivo*

#### Animals and Experimental Design

The experimental study followed the guidelines for the care and use of laboratory animals for the assessment of antioxidative health function, which was part of the food inspection and evaluation technical standards (2012 edition). The experimental animals were 60 healthy male Kunming mice (22 ± 2 g), which were obtained from the Zhejiang Academy of Medical Sciences. Ethics approval for this research was obtained from the committee of college of forestry and biotechnology, Zhejiang A & F University which is the University’s authority responsible for providing ethics approval for research involving animals. No further approval was required as per the University’s guidelines and national regulations. Animals were maintained under standard conditions (temperature 22 ± 1°C, humidity 45–55%, 12 h light per day). The mice were fed adaptively for 1 week, and randomly assigned into six groups (10 in each group). The animals were given *ad libitum* access to food and water. Animals in the five experimental groups were injected once daily with D-galactose (1.2 g kg^-1^, injection volume 0.1 mL⋅10 g^-1^) in hypodemic of hackles for 6 weeks, while animals in the normal control (NC) group were administered normal saline. For the VC positive control group (PC), mice were irrigated with VC solution (100 mg kg^-1^ day^-1^), which was dissolved in normal saline. Mice in the treatment groups were fed with extraction of *A. roxburghii* at different doses. The treatment groups were divided into three groups: (1) high dose (HD) group: 200 mg kg^-1^ day^-1^; (2) medium dose (MD) group: 100 mg kg^-1^ day^-1^; (3) low dose (LD) group: 50 mg kg^-1^ day^-1^. In addition, the NC group and model control (MC) group used the same method to get an equal volume of saline solution. In the meantime, continued to offer D-galactose except normal group, for 6 weeks.

#### Biochemical Assays

Twenty-four hours after the final drug administration, all mice were fasted. Blood samples were collected by enucleation and centrifuged at 4000 rpm for 10 min to give the serum. The mice were killed by cervical dislocation the liver tissue was quickly removed, washed, and homogenized in ice-cold physiological saline to prepare a 10% (w⋅v^-1^) homogenate. The homogenate was centrifuged at 4000 rpm for 10 min to remove cellular debris, and the supernatant was collected for analysis. The levels of T-SOD, GSH-Px and MDA levels were determined following the manufacturer’s instructions.

### Identification of Phenolic Compounds

The stock solutions were appropriately diluted to prepare a series of standard working solutions. The solutions were brought to room temperature and filtered through 0.22 μm membrane filters before qualitative analysis. The identification of total phenolics of *A. roxburghii* was carried out using an ACQUITY Ultra Performance LC system equipped with a mass detector G2 Q-TOF micro mass spectrometer (Waters, Manchester, United Kingdom) equipped with an electrospray ionization (ESI) source operating in negative mode. Separations of individual phenolic compounds were performed on a UPLC BEH C_18_ column (100 mm × 2.1 mm, 1.7 μm) at 30°C. The mobile phase consisted of solvent A (0.5% acetic acid) and solvent B (100% acetonitrile). A gradient elution procedure was used: 0–10 min, 5–10% B; 10–18 min, 10–80% B; 18–24 min, 80–5% B. The flow rate was kept at 0.2 mL min^-1^, and the injection volume was 2 μL. The nebulizer gas was set to 500 L h^-1^ at a temperature of 350°C under negative ion mode. The cone gas was set to a flow rate of 50 L h^-1^, and the source temperature was set to 120°C. The capillary voltages were set to 2.5 kV, and the cone voltages were set to 15 V. The TOF data being collected between m/z 250 and the MS/MS experiments were performed using high collision energy of 45 V for fragment ion information. The composition for the precursor ions and for the fragment ions were calculated using the MassLynx 4.1 software incorporated in the instrument.

### Statistical Analysis

The data were expressed as mean ± standard error. Significant difference was assessed by analysis of variance (ANOVA) and the Bonferroni multiple comparison test using SPSS (Statistical Package for the Social Science) statistical software (version 17.0, SPSS, Chicago, IL, United States).

## Results

### Modeling and Fitting the Model

The experimental design and corresponding response data for the content of total phenolics from *A. roxburghii* are presented in **Table 1**. Design Expert Software was used to plan the test and choose extraction time (*A*), microwave power (*B*), solid–liquid ratio (*C*), and alcohol concentration (*D*) as independent variables and the extraction yield of polyphenols from *A. roxburghii* (*Y*) was used as the response value (**Table 1**). The number 1–24 is factorial experiment. Quadratic regression analysis of data in the table, regression equation is obtained as follows (Khuri and Mukhopadhyay, 2010).

**Table 1:** Experimental design and results for microwave extracting of *Anoectochilus roxburghii.*

Run	Microwave time	Microwave power	Solid–liquid ratio	Ethanol concentration	Total phenolics yield (%)
1	20	200	50	70	0.9047
2	50	200	50	70	1.0440
3	20	400	50	70	0.9674
4	50	400	50	70	1.1457
5	20	200	70	70	0.9024
6	50	200	70	70	0.8754
7	20	400	70	70	0.8686
8	50	400	70	70	1.0373
9	20	200	50	80	0.9867
10	50	200	50	80	1.0477
11	20	400	50	80	0.9481
12	50	400	50	80	1.1939
13	20	200	70	80	0.9698
14	50	200	70	80	1.1047
15	20	400	70	80	1.0643
16	50	400	70	80	1.1924
17	5	300	60	75	0.6743
18	65	300	60	75	0.8038
19	35	100	60	75	0.7067
20	35	500	60	75	0.8408
21	35	300	40	75	0.7024
22	35	300	80	75	0.7510
23	35	300	60	65	1.2476
24	35	300	60	85	1.0791
25	35	300	60	75	1.1840
26	35	300	60	75	1.1898
27	35	300	60	75	1.1551
28	35	300	60	75	1.1262
29	35	300	60	75	1.1666
30	35	300	60	75	1.1898

$$\begin{array}{c} \begin{array}{l} Y=1.17+0.053A+0.053B-0.015C+0.030D+0.026AB\; \end{array}\\ \begin{array}{c} \begin{array}{l} -0.024AC+0.0067AD+0.013BC+0.00004575BD \end{array}\\ \begin{array}{l} \;+0.023CD-0.070A^{2}-0.074B^{2}-0.066C^{2}+0.037D^{2} \end{array} \end{array} \end{array}$$

The absolute values of coefficients directly reflect the impact of each factor on the response value from the equation, while the sign (positive or negative) of the coefficient reflects the orientation. The formula shows that time and microwave power has an equally strong effect on the extraction of total phenolics from *A. roxburghii*. Solid–liquid ratio has less effect, while the ethanol concentration has the least effect (Zheng et al., 2009).

### Validity of the Regression Model and Significance

The total phenolics yield was expressed as the percent ratio. It can be seen from **Table 1** that the measured polyphenols yields varied from 0.6743 to 1.2476% for the 30 experiments. The design matrix and the ANOVA results for the quadratic regression equation, and the influence of the various factors on the yield of total phenolics (**Table 2**). The model *F*-value of 3.11 implies the model terms are highly significant. There is only a 1.85% chance that a “model *F*-Value” this large could occur due to noise. According to the *P*-value, factors *A*^2^, *B*^2^, *C*^2^ are very significantly different (*P* < 0.01), the *A* is significantly different (*P* < 0.05). In addition, the variance of the quadratic regression model yielded a determination coefficient (*R*^2^) of 0.7435 and the coefficient of variation (CV) reflects the confidence level of the model was 11.82%. These values indicate the polynomial equation is generally adequate to describe the experimental results. “Adeq Precision” measures the signal to noise ratio. A ratio greater than 4 is desirable. The Adeq Precision of 6.771 indicates an adequate signal. This model can be used to navigate the design space (Liyana-Pathirana and Shahidi, 2005; Lin et al., 2015).

**Table 2:** Analysis regression model of relationship between variables and independent variable.

Source	Sum of squares	DF	Mean squares	*F-*value	*P*-value
Model	0.60998	14	0.60998	3.10646	0.0185
A	0.069184	1	0.069184	4.932716	0.0422
B	0.030107	1	0.030107	2.14654	0.1635
C	0.000665	1	0.000665	0.047448	0.8305
D	0.007511	1	0.007511	0.535507	0.4756
AB	0.010616	1	0.010616	0.756903	0.3980
AC	0.003026	1	0.003026	0.215757	0.6490
AD	0.000759	1	0.000759	0.054097	0.8192
BC	9.44E-05	1	9.44E-05	0.006732	0.9357
BD	3.16E-07	1	3.16E-07	2.25E-05	0.9963
CD	0.017793	1	0.017793	1.268605	0.2777
A^2^	0.174924	1	0.174924	12.47178	0.0030
B^2^	0.138992	1	0.138992	9.909845	0.0066
C^2^	0.188694	1	0.188694	13.45353	0.0023
D^2^	0.018848	1	0.018848	1.343822	0.2645
Residual	0.210384	15	0.210384		
Pure error	0.00312	5	0.00312		
Cor total	0.820364	29			

### Analysis of Response Surfaces

**FIGURE 1:**
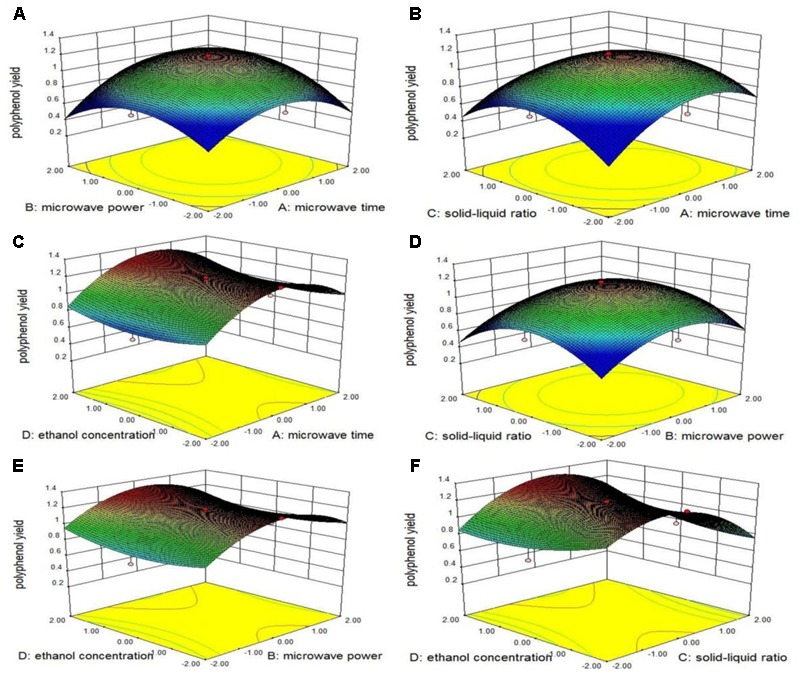
Response surface plots show the interaction among extraction time, microwave power, solid–liquid ratio, alcohol concentration and their effect on the extraction yield of *A. roxburghii*.

To illustrate the interactive effects of the independent variables and their mutual interaction on the extraction of total phenolics, three-dimensional response surface profiles of multiple non-linear regression models were plotted (**Figure 1**). **Figures 1A–C** depicts the interactions between the ethanol concentration and each of the other three factors based on the recovery of total phenolics. **Figure 1A** shows the extraction yield of total phenolics initially increased with increased extraction time and microwave power, and then decreased. The two curves have the same trend, and the contour lines show that these two factors have the same impact on total phenolics yield. Microwave power directly influences the molecular disruption, and allows total phenolics to dissolve quickly. Excessive microwave power will cause the extraction to overheat and cause phenolic compounds to decompose, thereby lowering the rate of total phenolics extraction. Prolonged microwave treatment is likely to damage total phenolics structure, and this would also reduce the extraction yield of total phenolics. **Figure 1B** shows that when the solid–liquid ratio was not constant, the total phenolics ratio increased over time and then dropped slightly, characterized by steep surfaces. When extraction time did not change, the total phenolics rate first increased and then decreased with increasing solid–liquid ratio, which gave a gentle performance curve. When the solid–liquid ratio was too high, the concentration of total phenolics gradient dropped so that there was little driving force for total phenolics to go into solution. The contours show that altering the extraction time is more efficient than altering the solid–liquid ratio to improve the extraction yield. When the ethanol concentration increased from 65 to 85% (**Figure 1C**), the extraction yield increased gradually as the extraction time increased, and reached a peak at about 40 min. Beyond this time, the yield began falling. This yield trend is attributed to increased extraction time. **Figure 1D** shows that adjustment of microwave power is more efficient than adjusting the solid–liquid ratio in optimizing extraction total phenolics extraction. **Figures 1E,F** shows that the influence of microwave power and solid–liquid ratio were more obvious than ethanol concentration. The yield of total phenolics increased with alcohol concentration, and reached a peak which the microwave power was 300–400 W and the solid–liquid ratio ranged between 1:50 and 1:60, after which the yield began to fall.

### Verification Experiments

The quadratic regression modes to predict that the optimal extraction conditions for MAE of *A. roxburghii* were: time, 37.55 min; microwave power, 320 W; solid–liquid ratio, 1:65.50; ethanol concentration, 83.90%. The theoretical total phenolics extraction yield under these conditions was 1.31%. These conditions were also used to predict the values of the responses using the model equation. The mean extraction yield of polyphenols was 1.31%, which is in good agreement with the predicted value, and confirms the adequacy of the model (Maran et al., 2013; Mat Nor and Arof, 2016).

### Antioxidant Activity *In Vitro*

**FIGURE 2:**
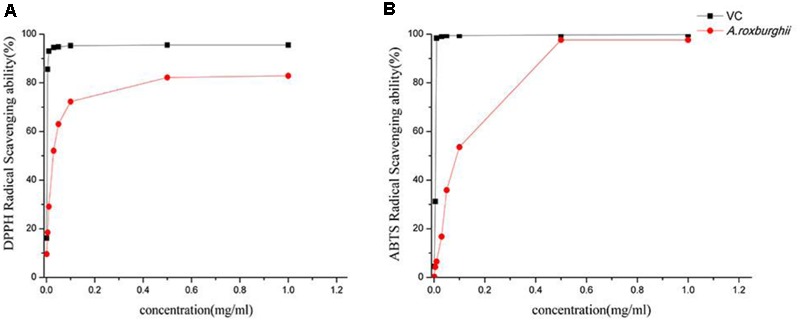
Scavenging capacity of DPPH **(A)** and ABTS **(B)**.

The scavenging capacity of *A. roxburghii* extract on DPPH free radical had been showed in **Figure 2A**. At the same concentration, the VC solution has more scavenging power against the DPPH free radical than the total phenolics solution of *A. roxburghii*, although both showed increased activity with increased concentration. The DPPH scavenging activity of VC reached 93.04% at the concentration of 0.01 mg mL^-1^, while the activity reached 95.52% at the concentration of 0.50 mg mL^-1^. The scavenging capacity of *A. roxburghii* increased rapidly up to 0.10 mg mL^-1^, but then increased more slowly. The clearance rates were 82.19 and 82.58% when the concentrations were 0.50 and 1.00 mg mL^-1^, respectively, indicating a balance point.

The total phenolics solution of *A. roxburghii* and VC all can clear the ABTS free radical which is shown in **Figure 2B** (Aprotosoaie et al., 2015) the solutions showed strong antioxidant activity, and radical clearance increased with increasing concentration. The antioxidant capacity of VC increased rapidly to 98.38% at a concentration of 0.01 mg mL^-1^, whereas the ABTS^+^ clearance rate of the *A. roxburghii* polyphenol solution rose relatively slowly. At 0.5 mg mL^-1^, the clearance reached 97.63% and later achieved equilibrium. The antioxidant level of *A. roxburghii* polyphenols was very close to that of VC until it reached equilibrium.

### Antioxidant Activity *In Vivo*

**FIGURE 3:**
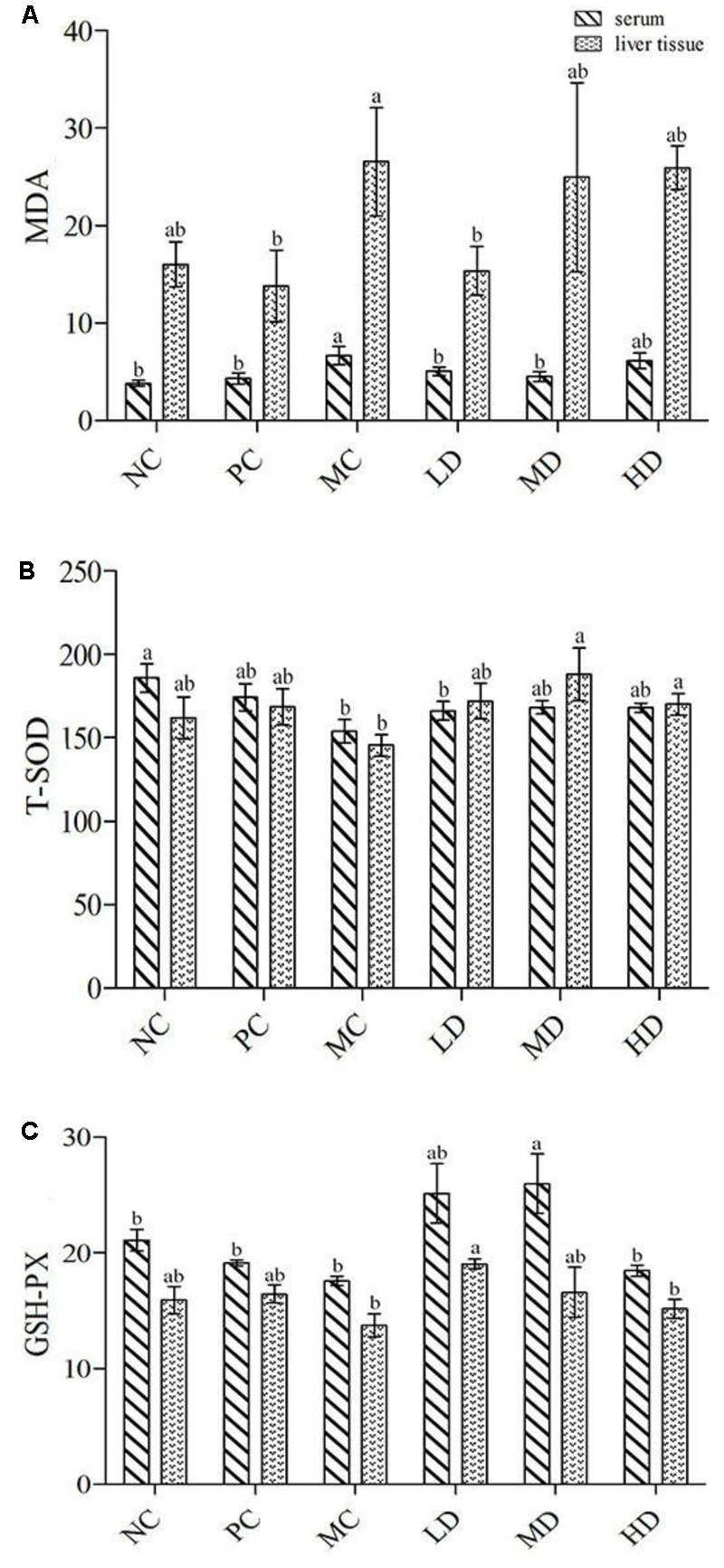
The value of **(A)** MDA, **(B)** T-SOD, and **(C)** GSH-P_X_ from serum and liver tissue.

The total phenolics extraction of *A. roxburghii* had some effect on serum and liver tissues of mice, as reflected by the activities of T-SOD and GSH-Px and the content of MDA (**Figure 3**). The content of MDA was significantly increased, and the activities of T-SOD and GSH-Px were obviously reduced, indicating that some lipid molecules had been attacked by free radicals. At the same time, the antioxidant enzymes had been consumed because of the excessive production of free radicals. While this result illustrated the validity of the oxidative damage model, compared with the model group, three different dose groups of total phenolics extraction from *A. roxburghii* all reduced the content of MDA in serum and liver tissue. In particular, the medium-dose and low-dose groups showed significant drops, and the enzyme activity of T-SOD and GSH-Px showed large increased. The MDA content reached 6.68 nmol mL^-1^ in serum while the low-dose and medium-dose groups reached 4–5 nmol mL^-1^. The MDA content reached 26.53 nmol mL^-1^ in liver tissue while the low dose-group reached 15.34 nmol mL^-1^. Each dose increased the activities of T-SOD and GSH-Px to different levels in serum and liver tissue. The T-SOD activity of liver tissue in the medium-dose group reached 188.05 U mL^-1^. The GSH-Px activities for the low-dose and medium-dose groups were all more than 25 U mL^-1^ in serum, and the activity in liver tissue for the low-dose group was stronger than the others (19.03 U mL^-1^). These results indicated that the total phenolics extracted yield could improve the antioxidant ability in mice. The extract showed significant antioxidant activity in the body, as indicated by MDA and GSH-Px, particularly for the low-dose group. The results for T-SOD suggest that the middle-dose group was the best choice, but there results between the low-dose and middle-dose groups for T-SOD in the liver were not obviously different. For the high-dose group, the T-SOD and GSH-Px activities were lower in serum and liver homogenate and MDA content was higher than in the other two dose groups.

### Identification of Phenolic Compounds

**FIGURE 4:**
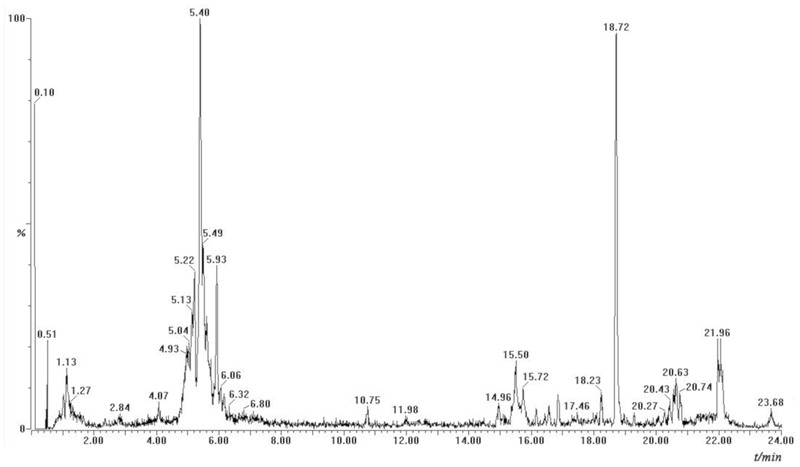
UPLC mass chromatogram in negative ion modes.

**FIGURE 5:**
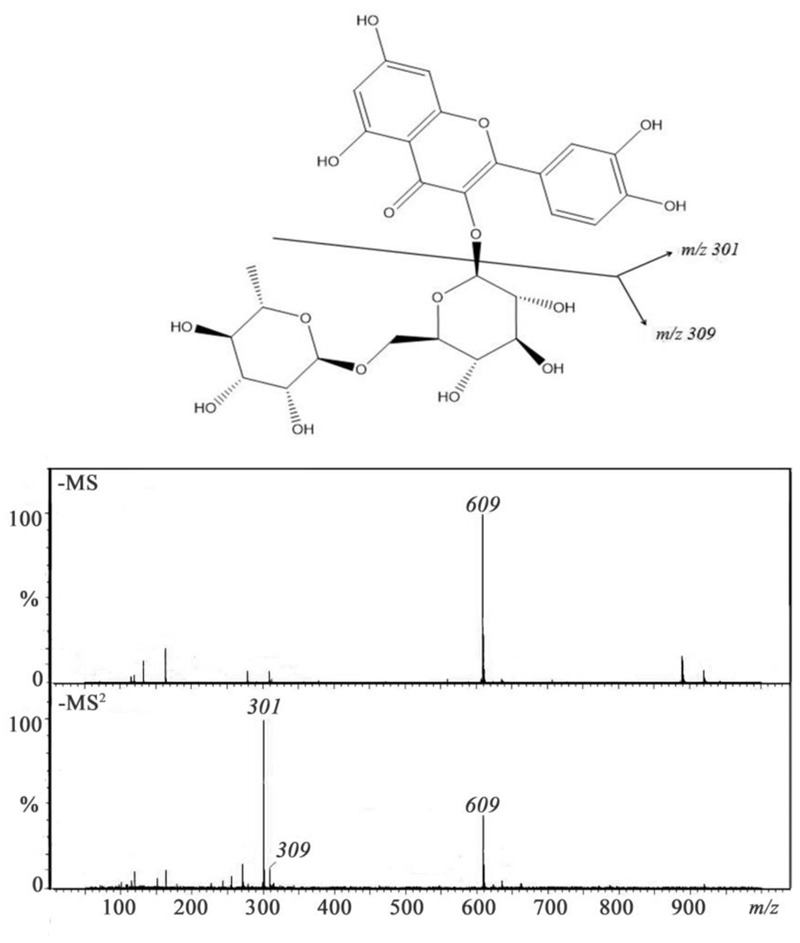
Mass spectra (MS and MS^2^) of rutin and possible fragmentation pathway.

UPLC systems however are designed to use columns with smaller particle sizes at higher pressures while Q-TOF is superior to make mass measurement with accuracy below 5 ppm for small molecules, thus facilitating greater resolution, sensitivity and speed when analyzing extracts from *A. roxburghii*. The identification and characterization of compounds was done by comparison of their retention times, UV spectrum and MS/MS data with isolated reference compounds and information available in literature. The phenolic compounds of *A. roxburghii* were characterized in negative ion modes. The negative ion mode of MS/MS analysis of extracts of *A. roxburghii* showed deprotonated [M-H]^-^ molecules (**Figure 4**). The identified phenolic compounds of *A. roxburghii* were listed in **Table 3**. **Figure 5** shows a representative example of the identity of phenolic compounds confirmed based on MS^2^ fragmentation pattern (rutin fragmentation pathway in negative ion modes). MS data revealed a molecular ion at 609 m/z, and MS^2^ data showed two fragment ions at m/z 301 [M-C_12_H_21_O_9_]^-^ and 309 [C_12_H_21_O_9_]^-^, which represented the quercetin and rutinose fragmentations of compound, respectively. The compound was identified as rutin by comparison to the authentic standard. In the present work, 21 phenolic compounds have been identified in *A. roxburghii* by using the combination of MS and MS/MS data and the information previously reported in the literature. The phenolic compounds such as rutin, quercetin, gallic acid, chlorogenic acid, and kaempferol detected in *A. roxburghii* are widely accepted as medicinally important compounds (Dai and Mumper, 2010). From the result, *A. roxburghii* possesses medicinally important polyphenols.

**Table 3:** The components identified by UPLC-Q-TOF-MS/MS in *A. roxburghii*.

Peak no.	Rt (min)	UV (nm)	Proposed	[M-H]^-^	MS/MS fragments	Identified	Reference
		λ_max_	formula	(m/z)	(m/z)	compounds	
1	3.03	270	C_7_H_6_O_5_	169.1450	125	Gallic acid	Tang et al., 2016
2	3.48	325	C_9_H_8_O_4_	179.1005	135	Caffeic acid	Rameshkumar et al., 2013; Fang et al., 2015
3	4.02	320	C_16_H_18_O_9_	353.1610	191, 179	Chlorogenic acid	Rameshkumar et al., 2013
4	5.05	278	C_15_H_14_O_6_	288.9121	123	L-Epicatechin	Li et al., 2006
5	5.13	353, 252	C_21_H_20_O_12_	463.0870	300	Hyperoside	Chen et al., 2012; Deng et al., 2013
6	5.61	279	C_9_H_8_O_3_	163.0396	119, 93	*p*-Coumaric acid	Kumar et al., 2015; Kolniak-Ostek, 2016
7	5.85	354, 254	C_27_H_30_O_16_	609.1456	301	Rutin	Jiménez-Sánchez et al., 2016; Kolniak-Ostek, 2016
8	6.03	310, 290	C_10_H_10_O_4_	193.0499	178	Ferulic acid	Jeyadevi et al., 2013
9	6.09	354, 254	C_21_H_20_O_12_	463.0870	301, 257, 179, 151	Isoquercitrin	Simirgiotis et al., 2013
10	6.55	254	C_28_H_32_O_16_	623.1619	315	Narcissin	Jiménez-Sánchez et al., 2016
11	6.68	254	C_21_H_20_O_11_	447.0916	357, 327, 297, 285	Orientin	Li et al., 2006; Bilia et al., 2008
12	6.83	354, 254	C_22_H_22_O_12_	477.1021	314	Isorhamneti*n*-3-*O*-glucoside	Li et al., 2016
13	6.90	355, 254	C_21_H_20_O_11_	447.0927	301, 179, 151	Quercitrin	Simirgiotis et al., 2013
14	6.93	355	C_21_H_20_O_12_	463.0870	301	Quercetin-3-*O*-β-D-glucoside	Kolniak-Ostek, 2016
15	7.11	349, 255	C_21_H_20_O_11_	447.0926	285	Luteoloside	Fang et al., 2015
16	7.15	351	C_21_H_20_O_11_	447.0939	161	Kaempferol-3-*O*-galactoside	Guo and Zhang, 2015
17	8.48	360, 256	C_15_H_10_O_7_	301.0349	179, 151, 107	Quercetin	Kumar et al., 2015; Jiménez-Sánchez et al., 2016
18	8.85	340, 258	C_15_H_10_O_5_	269.0442	225, 117	Apigenin	Lai et al., 2016
19	9.67	350, 254	C_15_H_10_O_6_	285.0395	257	Luteolin	Sánchez-Vioque et al., 2013
20	9.70	287, 230	C_15_H_10_O_6_	285.0395	151, 133	Kaempferol	Kumar et al., 2015
21	9.91	354, 265	C_16_H_12_O_7_	315.0502	300, 179, 151	Isorhamnetin	Simirgiotis et al., 2013

## Discussion

To maximize the extraction of active ingredients from plants, the choice of extraction method is very important. In recent years, considerable efforts have been made by researchers from diverse backgrounds to optimize the extraction process (Pan et al., 2003). At present, MAE, a new extraction technology that combines microwave treatment with traditional extraction methods, is widely used to extract the active ingredients in plants (Jafarifar et al., 2005). On a macroscopic level, microwave energy penetrates evenly into the extraction mixture, and, microscopically, microwave heating generates an electromagnetic field that can accelerate the diffusion of analytes and increase the efficiency of the extraction (Chen et al., 2015). The four factors were extraction time (5–65 min), microwave power (100–500 W), solid–liquid ratio (1:40 to 1:80), and ethanol concentration (65–85%) which could reduce the extraction cost and time that compared with the traditional extraction method.

Expected the extraction method, the choice of analysis methods is also crucial. RSM is applied to each neighborhood as a new analysis method to extract the flavonoids, polyphenols, and polysaccharides in plants or herbs. Therefore, it had been found by some test and verify that the response surface methodology is a highly accurate and good optimization effect, which is suitable for popularization and application in the optimization of process parameters. Some research both at home and abroad has attempted to optimize the extraction of *A. roxburghii*, to obtain the effective components, but mainly through orthogonal experimental design rather than RSM (Zhao et al., 2008; Zeng et al., 2016). Some studies on optimization of *A. roxburghii* have used RSM for aqueous extraction, but studies on ethanol extraction have not been reported. Orthogonal test design focused on how to arrange reasonably and to take into account several factors to determine the best combination, but a clear regression over a wide range of conditions is not achieved. Only discrete data can be analyzed and accuracy and predictability are not high. This study only need 30 experimental groups to reach a conclusion that the optimum extraction conditions for MAE of *A. roxburghii* are: time, 37.55 min; microwave power, 320 W; solid–liquid ratio, 1:65.50; ethanol concentration, 83.90%, and the total phenolics extraction yield is 1.31%.

Researchers have performed preliminary studies on the chemical compounds of *A. roxburghii*, including phenolic, polysaccharides, triterpenoids, amino acids, etc. In our preliminary study, we confirmed that *A. roxburghii* was rich in phenolic compounds. Phenolic compounds are considered to have many functions, such as removing active oxygen, preventing hemal sclerosis, improving nutrition for tissue, antiaging and preventing aging dementia. However, limited studies explored the mechanism by which the compounds regulate the antioxidant defense system *in vivo* and *in vitro.*

In this experiment, antioxidant activity *in vitro* was determined by DPPH and ABTS methods. DPPH free radical is commonly used to screen free radical scavengers and evaluate the antioxidant capacity (Singh et al., 2016). The electronic structure of DPPH free radical gives a dark color in ethanol solution, but combination with a lone electron of an antioxidant lightens the color significantly. Accordingly, the degree of color change can be used to assess the antioxidant activity. Determine the absorbance which showed a strong antioxidant activity, when the concentration of *A. roxburghii* was 0.5 mg mL^-1^ the clearance rate was reached 82.19%. DPPH method is widely used in the determination of antioxidant activity *in vitro*, and in some article we could find that *Cordyceps mycelium* scavenging effect on DPPH only reached 70% when the concentration of alcohol extraction was 16 mg mL^-1^ (Li et al., 2010). Another research reported that *Ganoderma lucidum* extraction on the scavenging rate of DPPH radical; it can be seen that when the concentration was 0.5 mg mL^-1^, the scavenging capacity was 60%, and when the concentration was reached 1 mg mL^-1^, the scavenging capacity was more than 80%. Even when the concentration of *Ganoderma atrum* was reached 1 mg mL^-1^, it reached more than 90% (Gong et al., 2006). The extract from *A. roxburghii* although did not reach more than 90% of the removal rate, but compared with these precious medicinal materials, the antioxidant capacity of *A. roxburghii* also has broad prospects. ABTS is one of the common antioxidant indexes *in vitro*, and the principle is that ABTS^+^ will produce a stable free radical with a bluish-green color in an oxidizing environment. After combination with radical scavenger, the solution color weakens, and the decline in color can be used to evaluate antioxidant activity against ABTS^+^ free radical. We can see from the results that ABTS^+^ exhibited stronger scavenging activity which has reached the level of 97.63% when the concentration of 0.5 mg mL^-1^. So the antioxidant capacity of the extract of *A. roxburghii* need further study and deep measurement.

MDA, T-SOD, GSH-Px are the main index *in vivo* antioxidant assay. MDA is a lipid peroxide that is formed by enzymatic and non-enzymatic reaction to produce oxygen free radicals to attack polyunsaturated fatty acids and cause the lipid peroxidation in the body. The content of MDA can reflect the degree of lipid peroxidation in the body and can be used to infer the degree of damage to the cellular structure (Liao et al., 2014). T-SOD plays a very important role in regulating the balance of *in vivo* oxidation. This enzyme can protect cells from free radicals and its activity is an indicator of the body’s antioxidant level. This also provides a measure of aging and closeness to death (Cui et al., 2013). GSH-Px exists in many organisms to catalyze the decomposition of hydrogen peroxide in the body and thereby prevent damage to cell membrane. This enzyme plays an important role in maintaining cell viability (Shen et al., 2016). In the previous study, the method has been determined the antioxidant *in vivo* only for water extraction, alcohol extraction has not been studied (Cui et al., 2013). Therefore, this method is adjusted and modified according to its method and other related methods for *in vivo* antioxidant assay. This paper results were similar to other studies that the higher of MDA content, the more serious of injury in mice, and the higher T-SOD and GSH-Px value showed that the mice had improved oxidative damage in some extent. So it could be found in this study that the low and medium dose groups of *A. roxburghii* had improved oxidative stress condition. On the other hand, the high dose group may have increased damage due to excessive blood concentration or excess antioxidants (Abdelgawad et al., 2016). Therefore, our body needs a small amount of antioxidants, should not be too much.

This is the first report on phenolic compounds detected by UPLC-Q-TOF-MS/MS analysis for *A. roxburghii.* In the present work, 21 phenolic compounds had been identified in *A. roxburghii.* Compared with other analytical methods, possible active substances were obtained more quickly (Singh et al., 2016). Due to the lack of relevant standard products, other substances mainly speculate the possible structure by the fragmentation law of ion fragments of the one level and two levels mass spectra. The results obtained can provide a reference and direction for further research on oxidized monomers.

## Conclusion

The RSM is utilized to optimize the process condition on yield of MAE are 37.55 min, 320 W, 1:65.5, and 83.90%, and the total phenolics yield was 1.31%. DPPH maximum clear rate reached 82.58% and ABTS^+^ was 97.63% which could be drawn a conclusion that *A. roxburghii* had a strong antioxidant impression. Serum and liver homogeneous (10%) of mouse were detected as MDA, T-SOD, and GSH-P_X_ the middle and low-dose groups had high ability to clear free radicals. In short, total phenolics extracted from *A. roxburghii* showed a strong antioxidant activity both in chemical simulation system and *in vivo–in vitro* assays systems. An improved method utilizing UPLC-Q-TOF-MS/MS was developed for the simultaneous identification the compound in *A. roxburghii*. The developed method identified 21 phenolic compounds in *A. roxburghii*. This could provide reference for the development of *A. roxburghii* as food additives and nature antioxidants in the future.

## Compliance with Ethical Standards

All applicable international, national, and institutional guidelines for the care and use of animals were followed. This article does not contain any studies with humans performed by any of the authors.

## Author Contributions

The first author MX mainly responsible for the operation of the whole experiment and the writing of the paper. SY, SL, and YL mainly responsible for assisting the first author in the experiment. QS, MD, and MW mainly responsible for providing technical guidance and experimental materials.

## Conflict of Interest Statement

The authors declare that the research was conducted in the absence of any commercial or financial relationships that could be construed as a potential conflict of interest.
